# 25-OH vitamin D threshold for optimal bone mineral density in elderly patients with chronic kidney disease

**DOI:** 10.3389/fragi.2022.1026663

**Published:** 2022-10-21

**Authors:** Debajyoti Roy, Chee Yong Ng, Zheng Xi Kog, Wenxiang Yeon, Cheng Boon Poh, Sreekanth Koduri, Chang Yin Chionh, Rehena Sultana, Troy Hai Kiat Puar

**Affiliations:** ^1^ Changi General Hospital, Department of Renal Medicine, Singapore, Singapore; ^2^ Centre for Quantitative Medicine, Duke-NUS Medical School, Singapore, Singapore; ^3^ Changi General Hospital, Department of Endocrine, Singapore, Singapore

**Keywords:** chronic kidney disease, vitamin D, hip fracture, bone mineral density, elderly

## Abstract

**Introduction:** Vitamin D deficiency is common in chronic kidney disease (CKD) and is associated with lower bone mineral density (BMD), decreased muscle strength, and increased hip fracture risk. Guidelines have suggested targeting 25-OH vitamin D (25(OH)D) levels between 20 and 30 ng/ml. However, vitamin D metabolism is altered in CKD, and threshold levels for optimal BMD are unknown.

**Methods:** We included 1097 patients with hip fractures. CKD was defined as estimated glomerular filtration rate <60 ml/min/1.73 m (Mucsi et al., Clin. Nephrol., 2005, 64(4), 288–294) and low BMD defined as T score ≤ −2.5 at femoral neck. We assessed the association of 25(OH)D with low BMD in patients with and without CKD: using the conventional threshold 25(OH)D < 30 ng/dl, as well as a new threshold.

**Results:** CKD was present in 479 (44%) patients. Using a threshold of 25(OH)D < 30 ng/ml, there were no significant differences in patients with CKD and low BMD when compared to the other groups. We identified 27 ng/ml as a better threshold with the Youden index. Using 25(OH)D < 27 ng/ml as a threshold, 360 of 482 patients (74.7%) with low 25(OH)D had low BMD, compared to only 185/276 (67%) of patients with adequate vitamin D, *p =* 0.02, which was irrespective of the presence or absence of CKD. Furthermore, patients with CKD and 25(OH)D < 27 ng/ml had a higher odds ratio of mortality upon follow-up, 1.61, 95% CI: 1.08–2.39, compared to those with CKD and 25(OH)D ≥ 27 ng/ml.

**Conclusion:** We find that 25(OH)D < 27 ng/ml is associated with low BMD in patients with and without CKD. Further prospective studies targeting vitamin D repletion to at least 27 ng/ml and the outcome of hip fractures will be useful to validate these findings.

## Introduction

Eighty percent of individuals with chronic kidney disease (CKD) have low vitamin D levels (Ngai et al., 2014). Meanwhile, low vitamin D levels are associated with low bone mineral density (BMD) and increased falls (Mucsi et al., 2005; Boudville et al., 2010; Leeho et al., 2014a). Hip fractures sustained in elderly patients with CKD are associated with a worse prognosis.

The 2017 KDIGO guidelines for CKD–MBD advise assessing bone mineral density in patients with CKD 3–5D. However, they do not make a decisive statement on the need for measuring 25(OH)D levels nor do they give any guidance on target levels to be achieved (Ketteler et al., 2017). A recent meta-analysis has examined the efficacy of vitamin D supplementation in reducing the risk of fracture and has demonstrated that higher blood 25(OH)D concentrations were associated with lower risks of hip fracture. An increase in 25(OH)D concentration of 10 ng/ml was associated with a 7% lower risk of any fracture and a 20% lower risk of hip fracture (Yao et al., 2019). Vitamin D deficiency has been variably defined but most guidelines accept <20 ng/ml as deficiency and 21–29 ng/ml as insufficiency (Holick et al., 2011). Although vitamin D supplementation is safe, optimal serum 25(OH)D target levels have not yet been defined, especially in the CKD population. This problem becomes imperative given that anti-resorptive therapies (i.e., bisphosphonates and denosumab) are not recommended in CKD with GFR <30–35 ml/min/1.73 m (Mucsi et al., 2005), and there is a well-known association of low vitamin D levels with osteoporosis (Prolia, 2011; FOSAMAX, 2012).

In this study, we report on the association of 25(OH)D levels and low bone mineral density in non-dialysis-dependent CKD.

## Aims

The primary aim of this study was to investigate the association between 25(OH)D deficiency and renal impairment (eGFR <60 ml/min/1.73 m^2^) on low BMD for elderly patients with hip fracture.

We further evaluated the 25(OH)D level at which low BMD (T score ≤ –2.5) was prevalent. The other factors that were evaluated are demographics, length of stay, and mortality in vitamin D-deficient patients with CKD.

### Methods

This was a retrospective observational study and was conducted with ethics approval from the SingHealth Institutional Review Board (2017/2962) at Changi General Hospital, Singapore. The study was performed in accordance with the SingHealth Institutional Review Board guidelines. The need for informed consent was waived by the SingHealth Institutional Review Board.

All patients aged 65 years or older admitted due to hip fracture between 1 August 2014 and 31 August 2016 were included. All patients were followed up from index date to 10 June 2018 or death, whichever came earlier. Patients with acute kidney injury and transient renal impairment were excluded.

The demographic data that we collected included estimated glomerular filtration rate (eGFR) and 25(OH)D levels from institutional electronic medical records. Serum 25(OH)D levels were assayed by radioimmunoassay (Roche Diagnostics, Switzerland). The measurement of BMD was at the femoral neck using dual energy X-ray absorptiometry (Hologic QDR Discovery Wi, United States).

The eGFR was estimated using the CKD-Epi formula (Levey et al., 2009) and vitamin D deficiency was defined as 25(OH)D ≤ 30 ng/ml (Holick et al., 2011). Low BMD was defined as T score≤–2.5 based on the World Health Organization criteria for osteoporosis (Kanis, 1994). Eligible patients were classified into two groups based on their eGFR status: CKD (eGFR<60 ml/min/1.73 m; Mucsi et al., 2005) and non-CKD (eGFR≥ 60 ml/min/1.73 m; Mucsi et al., 2005). They were further classified based on vitamin D deficiency and BMD status. Patients with missing data for serum creatinine, BMD, and 25(OH)D measurements were excluded.

In an earlier article, we had reported on mortality in a subset of these patients who had undergone surgery (Roy et al., 2021).

### Outcome

The primary outcome was BMD and was treated as binary data with low BMD (osteoporotic range: T score≤–2.5) *versus* non-osteoporotic range BMD (T score>–2.5). Secondary outcomes were in-hospital mortality and length of hospital stay.

### Statistical methods

Patients were categorized as vitamin D-deficient (25(OH)D ≤ 30 ng/ml) and non-deficient (25(OH)D > 30 ng/ml) groups. The primary objective of this study was to find an association between vitamin D deficiency and low BMD among non-dialysis-dependent CKD and normal kidney function patients. All of the demographic variables were summarized based on CKD and normal kidney function patients. Continuous variables were summarized as mean ± standard deviation (SD) or median (interquartile range (IQR)), whichever appropriate, and categorical variables were summarized as frequency (proportions). BMD in continuous scale was compared among composite exposure of 25(OH)D and CKD status using the Kruskal–Wallis test. A best cut off for the 25(OH)D level based on this cohort was determined using the Youden index for low BMD using univariate logistic regression (Schisterman et al., 2008). Quantitative association from logistic regression was represented as odds ratio (OR) with 95% confidence interval. Diagnostic accuracy of referenced and proposed cut off was also reported using sensitivity, specificity, positive predictive value (PPV), and negative predictive value (NPV), with corresponding 95% confidence interval (95%CI). We also calculated sensitivity and specificity for cut-off values of 25(OH)D at ≤ 30 ng/ml and ≤27 ng/ml for association with low BMD. All of the statistical analyses were performed using SAS v9.4 (SAS Institute Inc., NC, United States). Statistical significance was set at *p* < 0.05.

## Results

There were a total of 1097 patients with hip fracture, of which 497 had CKD (eGFR <60 ml/min/1.73 m^2^) and 678 had normal kidney function (non-CKD group). Table 1 describes the demographics of the two groups. The majority in the CKD group were stages 3 and 4 (439 of 479, 91.6%). Meanwhile, 25(OH)D levels were deficient in 44.0% and 39.5% in the CKD and non-CKD groups, respectively.

**TABLE 1:** Demographic characteristics of patients based on CKD status.

Characteristic	CKD (*n* = 479)	Non-CKD (*n* = 678)
Age (years)	82.5 (±7.64)	78.2 (±8.5)
Women	340 (71%)	399 (64.6%)
Race: Chinese	348 (74.4%)	459 (75.9%)
Vitamin D levels ≤30 ng/ml	211 (44%)	268 (39.5%)
CKD G3a	222 (47.3%)	—
CKD G3b	141 (30.1%)	—
CKD G4	76 (16.2%)	—
CKD G5	30 (6.4%)	—
Diabetes	298 (62.2%)	386 (62.5%)
BMD (T score) ^a^	–3.0 (1.06)	–3 (1.03)
Died	123 (25.7%)	124 (20.1%)
Duration from discharge to death (years)	2.3 (±0.98)	2.4 (±0.93)

^a^ (IQR).

**FIGURE 1:**
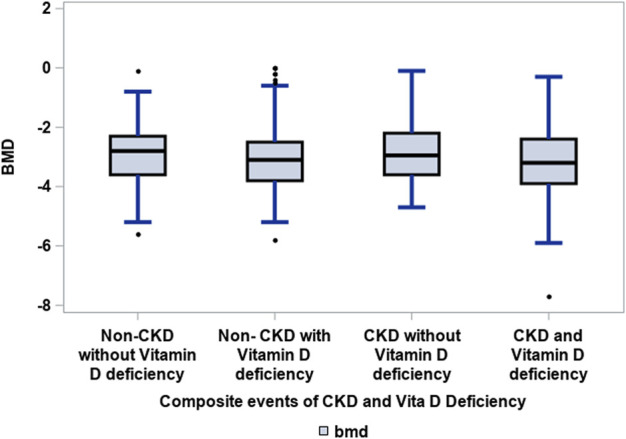
Association of CKD with vitamin D deficiency to low BMD amongst elderly patients with hip fractures. Groups: CKD and vitamin D deficiency *versus* CKD without vitamin D deficiency, non-CKD without and with vitamin D deficiency. Kruskal–Wallis test: 0.0510. Vitamin D deficiency was defined as 25(OH)D ≤ 30 ng/ml (Holick et al., 2011) and CKD was defined as eGFR<60 ml/min/1.73 m (Mucsi et al., 2005).

Patients with CKD and vitamin D deficiency, defined as 25(OH)D < 30 ng/ml, were not significantly associated with low BMD (T score ≤—2.5) at the femoral neck as compared to the groups: CKD without vitamin D deficiency, and non-CKD with vitamin D deficiency and without vitamin D deficiency (see Figure 1).

We identified a cut-off point of 25(OH)D at ≤ 27 ng/ml for association with low BMD (T score ≤ –2.5) using Youden’s index, irrespective of the presence or absence of CKD. The association of 25(OH)D levels ≤27 ng/ml with low BMD is described in Table 2. We found that this cut off was more sensitive to identify a greater risk of low BMD: a greater proportion of patients with 25(OH)D at ≤ 27 ng/ml had low BMD, 360 of 482, 74.7%, when compared to those with 25(OH)D at ≥ 27 ng/ml, 185 of 276, 67.0%, *p* = 0.02 (Table 3).

**TABLE 2:** Association of 25(OH)D level ≤27 ng/ml with low BMD.

25(OH)D	Low BMD		
	No	Yes	Total
No	66	144	210
Yes	147	401	548
Total	213	545	758

Chi-square *p* value = 0.02.

**TABLE 3:** Association 25(OH)D with low BMD at ≤30 ng/ml *vs*. ≤ 27 ng/ml.

a. Cut off at 25(OH)D level ≤30 ng/ml			
25(OH)D (≤30 ng/ml)	Low BMD		
	No	Yes	Total
No	91	185	276
Yes	122	360	482
Total	213	545	758

Statistics	Value (%)	95% CI
Sensitivity	66.06	61.91%–70.03%
Specificity	42.72	35.99%–49.66%
Positive predictive value	74.69	72.14%–77.08%
Negative predictive value	32.97	28.82%–37.41%
Accuracy	59.50	55.91%–63.02%

b Cut off at 25(OH)D level ≤27 ng/ml			
25(OH)D (≤27 ng/ml)	Low BMD		
	No	Yes	Total
No	66	144	210
Yes	147	401	548
Total	213	545	758

Statistics	Value (%)	95% CI
Sensitivity	73.58	69.66%–77.24%
Specificity	30.99	24.85%–37.66%
Positive predictive value	73.18	71.10%–75.15%
Negative predictive value	31.43	26.41%–36.92%
Accuracy	61.61	58.04%–65.09%

Mortality was higher in the CKD group with 25(OH)D levels ≤ 27 ng/ml as compared to those with 25(OH)D levels >27 ng/ml (OR 1.61(1.08, 2.39).

The length of hospital stay post-admission for fracture was longer in the CKD with vitamin D deficiency group by 1.5 days compared to the non-CKD group (see Table 4).

**TABLE 4:** Length of stay (days) in relation to the presence or absence of CKD and 25(OH)D levels.

Composite events of CKD and vit D deficiency	N	Mean	Median	25th pctl	75th pctl
Non-CKD without vitamin D deficiency	139	11.33	8.97	6.53	12.25
Non-CKD with vitamin D deficiency	296	12.01	9.10	6.64	13.11
CKD without vitamin D deficiency	128	12.68	10.22	7.04	15.67
CKD and vitamin D deficiency	195	13.94	10.48	7.59	15.48

*p* value (Kruskal–Wallis test): 0.003. Vitamin D deficiency was defined as 25(OH)D ≤ 30 ng/ml (Holick et al., 2011), and CKD was defined as eGFR<60 ml/min/1.73 m (Mucsi et al., 2005). Pctl, percentile.

## Discussion

In our analysis of this retrospective cohort of 1097 elderly patients with hip fracture, we report on the association of vitamin D deficiency with BMD in a CKD group *versus* a group with normal kidney function. We found that a 25(OH)D cut off ≤27 ng/ml was significantly associated with low BMD, irrespective of the presence or absence of CKD. In the CKD group, this cut-off value was also associated with odds of 1.61 for mortality in comparison to those without vitamin D deficiency. The group CKD with vitamin D deficiency also had a longer length of hospital stay. When using a 25(OH)D cut off <30, the group CKD with vitamin D deficiency was not significantly associated with a lower BMD in comparison to groups: CKD with no vitamin D deficiency, and non-CKD groups with and without vitamin D deficiency.

Vitamin D deficiency is common in CKD. Even in Asian countries with abundant sunlight, vitamin D deficiency is reported in up to 80% of patients. The KDIGO guidelines on CKD–MBD recognize the importance of vitamin D deficiency and “[suggest] that 25(OH)D levels might be measured and repeat testing determined by baseline values and therapeutic interventions” (Ketteler et al., 2017). Unfortunately, the frequency of testing, actionable values, and target levels to be achieved are not defined. Most authorities define vitamin D deficiency as < 20 ng/ml, while insufficiency ranges from 20 to 29 ng/ml. The optimal levels are not well defined, with various suggestions ranging from 20 to 30 ng/ml. In CKD, the low 25(OH)D levels are believed to be due to high levels of fibroblast growth factor-23 (FGF-23) that are secreted due to high phosphate levels, which suppress 1-alpha hydroxylase activity and induce 24-hydroxylase expression, and results in degradation of 1,25(OH)D. Michaud et al. (2010) suggest that in CKD high PTH itself may suppress liver CYP450 isoforms and hence decrease 25(OH)D synthesis. Whatever the mechanism, it is well-accepted that vitamin D deficiency is associated with secondary hyperparathyroidism, low BMD, and muscle weakness, which increases the risk of falls in the elderly CKD (Bataille et al., 2016; Leeho et al., 2014b; Mucsi et al. 2005). Given the importance of vitamin D repletion, it is imperative that a cut off be established for optimal bone and muscle health, especially in CKD patients. In our cohort, we found that 25(OH)D cut off at ≤ 27 ng/ml was associated with a greater prevalence of low BMD (T score ≤ –2.5), irrespective of the presence or absence of CKD. Boudville et al. (2010) similarly reported on 25 hemodialysis patients and found that 25(OH)D levels of 24–44 ng/ml was associated with maximal benefits with regard to muscle weakness and falls. Bataille et al. (2016) found a “dose–effect” relationship between 25(OH)D of >30 ng/ml and improved muscle strength assessed by hand grip in 130 elderly hemodialysis patients. Our findings make a strong case for a target 25(OH)D greater than 27 ng/ml for optimal bone and muscle health.

### Low 25(OH)D and mortality

We found in our group of elderly CKD patients with hip fracture that 25(OH)D levels ≤27 ng/ml were associated with odds of 1.61 for mortality as compared to those with higher levels. A meta-analysis of prospective studies addressing the relationship of low vitamin D levels and mortality risk in CKD including over 6853 patients reported that the mortality decreased by 14 percent for a 10 ng/ml increase in 25(OH)D levels (Pilz et al., 2011). In their meta-analysis of 20 observational studies, Zheng et al. (2013) investigated the effect of vitamin D supplementation on mortality risk and reported 26% lower mortality compared to those with no treatment. Patients with CKD have reduced 1-alpha hydroxylase activity, which is required for converting 25(OH)D to the active form 1,25(OH)D. The presence of extra renal 1-alpha hydroxylation has led nephrologists to consider use of nutritional vitamin D for replacement. However, the question of whether native vitamin D supplementation, or active vitamin D, or a combination of both should be recommended in CKD patients or ESRF remains unresolved because of the lack of randomized controlled trials (Bentata, 2019; Melamed and Thadhani, 2012).

### Low 25(OH)D and length of hospital stay

The CKD group with vitamin D deficiency had a longer length of hospital stay (median [10th, 90th centiles] 10.5 days (7.5 to 15.5) *versus* 9.0 days (6.5 to 12.3) for the non-CKD group with no vitamin D deficiency. Kim et al. (2016) examined the incidence of hip fracture, mortality and length of stay incorporating data from the USRDS. They similarly found that the non-dialysis group requiring CKD were associated with higher age standardized rates of hip fracture, with longer length of stay and resource utilization. This would translate to a higher health cost burden.

In CKD with vitamin D deficiency, there is currently a distinct preference for using synthetic vitamin D (i.e., calcitriol or alfacalcidol), despite it having no role in replacing deficiency. What this article adds to the current literature is that correcting 25(OH)D to a target level of >27 ng/ml with native vitamin D (i.e., colecalciferol or ergocalciferol) may help reduce the risk of hip fractures, not only by possibly improving BMD levels but also by improving muscle strength.

### Strengths and limitations

The strengths of our study are that it includes a relatively large patient group, with particularly elderly patients with osteoporotic fractures.

The limitations of our study include the retrospective design, which cannot demonstrate a causal-effect relationship between CKD, low vitamin D, and low BMD. In addition, a relatively small proportion of patients had CKD G5. Given that all of the patients were elderly and had already sustained a hip fracture, this may dilute the significance of vitamin D deficiency and low T scores. Furthermore, there were no intact PTH levels available, which might have been useful when assessing the effect of vitamin D deficiency on mortality.

## Conclusion

Osteoporotic fractures are common in patients with CKD and are associated with an increased risk of adverse clinical outcomes when compared to the non-CKD population. Using a 25(OH)D cut off ≤30 ng/ml, we did not find the combination of mild to moderate CKD with vitamin D deficiency to have lower BMD in comparison to the following groups: CKD with no vitamin D deficiency, or the non-CKD groups with and without vitamin D deficiency. At a 25(OH)D cut off ≤27 ng/ml, there was a significant association with T score ≤ –2.5, irrespective of the presence or absence of CKD. This cut-off value was also associated with odds for mortality of 1.61 and a longer length of stay by 1.5 days. These findings would make a strong case for a target 25(OH)D greater than 27 ng/ml for optimal bone and muscle health.

Further prospective studies are required to confirm the optimal level of 25(OH)D levels in the CKD population to improve bone health and muscular weakness.

## Data Availability

The raw data supporting the conclusion of this article will be made available by the authors, without undue reservation.
